# Microplastics research in Nepal: Present scenario and current gaps in knowledge

**DOI:** 10.1016/j.heliyon.2024.e24956

**Published:** 2024-01-22

**Authors:** Kishor Kumar Maharjan

**Affiliations:** a Department of Environmental Science, Tri-Chandra Multiple Campus, Tribhuvan University, Nepal; b Faculty of Environmental Management, Prince of Songkla University, Thailand

**Keywords:** Freshwater, Microplastic pollution, Nepal, Plastics, Polymer

## Abstract

The topic of microplastics has drawn considerable scholarly interest in recent times. The objective of this study is to provide an overview of the current state of microplastic pollution research in Nepal and to make future research recommendations. To achieve the objective, three popular databases (Web of Science, SCOPUS and Google Scholar) were used. The results showed that the current scenario for microplastic research in Nepal is in its early stage, which commenced in 2020. A total of six papers were recorded over the period from 2020 to 2023. The research conducted in the fields were rivers, lakes, snow, and sediments. Studies have provided evidence of the occurrence of microplastics in diverse aquatic ecosystems. Lakeshore sediments show concentrations of 100.5 ± 58.6 items/kg dry weight, while shoreline sediments of Phewa lake exhibit variability between 55 and 122.5 items/kg. The lake water in winter records 2.96 ± 1.83 Microplastics per Liter (MPs/L), river water indicates 202 ± 100 items/m^3^, and snow demonstrates 30 MP/L. In freshwater ecosystems, microplastics, specifically fibers, were found to be the prevailing type, while fragments were recorded in road dust. The study conducted in Nepal provided evidence of the presence of a wide range of polymers. The polymers encompassed polypropylene (PP), polyethylene (PE), polyethylene terephthalate (PET), polyamide, polystyrene (PS), and polyester. Microplastic research in Nepal, initiated in 2020, covered rivers, lakes, snow, and sediments. Diverse aquatic ecosystems reveal microplastic presence, emphasizing the need for continued study and awareness. Although extensive research has been carried out on the subject of microplastic contamination and its effects on various creatures on a global scale, an examination of the implications of microplastics on animals, plants, and humans in Nepal has not been found in any scholarly publications. There exists a noticeable deficit of research investigating the consumption of microplastics by human.

## Graphical abstract

**Figure:**
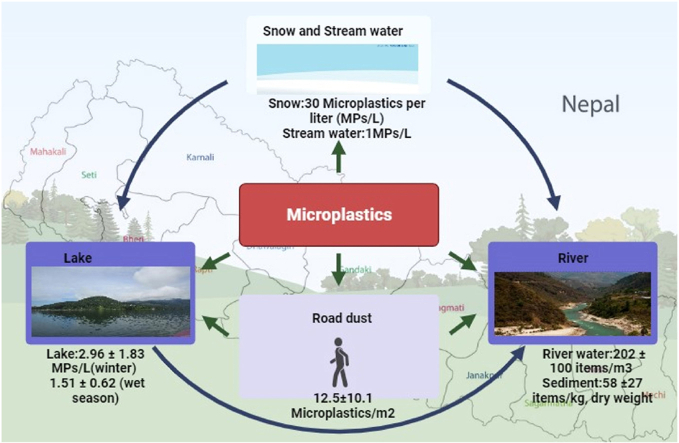

## 1 Introduction

Plastic is extensively utilized in every area of daily existence. In 2018, the worldwide manufacture of plastics approached a total of 360 million tonnes [1]. It is estimated that over 5 trillion plastic bags are manufactured on a global scale annually [2]. The average individual worldwide utilizes over 300 plastic bags each year [3]. The ongoing trend of rapid urbanization and economic growth has resulted in a global expansion in the production and use of plastic products. The World Bank published a report indicating that in 2016, around 242 million tonnes of plastic waste were produced, constituting 12 % of all municipal solid waste [4].

**Fig. 1:**
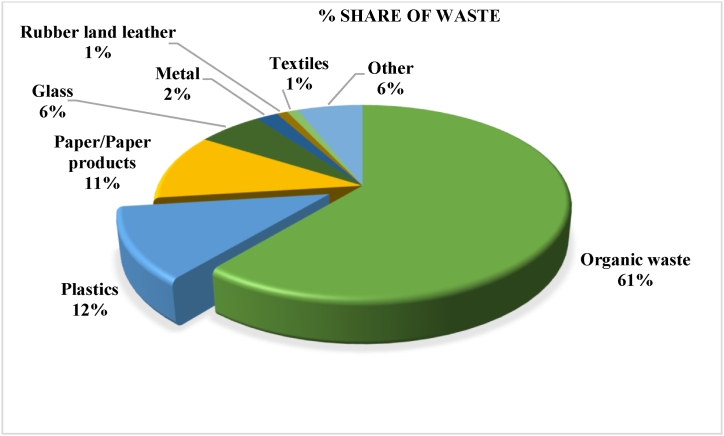
Composition of solid waste in Nepal.

In the context of Nepal, it has been observed that approximately 16% of the total urban waste consists of plastic materials. This amounts to a daily output of approximately 2.7 tons of plastic waste [5]. The study conducted by Pathak [6] revealed that plastic waste accounted for 12 % of the total municipal solid waste, while organic waste dominates, comprising 61 % (Fig. 1). In the fiscal year 2021/22, Nepal imported 380,000 tons of plastic, while domestic production was around 165,000 tons. With inadequate plastic management, approximately 20.7 kilo tons (9 % of total consumption) leak into the environment annually due to the absence of effective policies [7].

Since the early 1990s, Nepal has witnessed the use of heavy quantities of plastic and plastic bags, resulting in significant waste management and environmental challenges. The country is facing issues like inadequate source segregation, substandard waste collection services, and the prevalence of open dumping landfills Pathak [6]. The majority of plastic waste are either burned or deposited in landfills. Numerous challenges hinder proper plastic waste management, including a lack of awareness about improper disposal practices and the presence of limited recycling centers. Furthermore, challenging topography and difficult terrain in hilly and mountain regions add complexity to waste collection and disposal efforts.

Numerous initiatives have been undertaken to address plastic waste pollution in Nepal. Legal efforts to ban thin plastic began two decades ago and are undergoing multiple revisions. The Government of Nepal initially embarked on a significant move to ban plastics smaller than 20 × 35 inches and thinner than 40 μm on April 1, 2015, gaining momentum gradually. However, the aftermath of the catastrophic April 25, 2015 earthquake shifted the government's focus to recovery, temporarily sidelining this initiative. In a renewed effort, the government introduced the Action Plan for Ban on Plastic Bags in May 2022. This plan outlined four strategies, including preventing the import of thin plastic products, banning single-use plastic bags, providing grants for eco-friendly bag production, and promoting personal use of environmentally friendly bags during shopping. A notice in the Nepal Gazette officially prohibited the production, import, sale, distribution, and use of plastic bags under 40 μm nationwide. Existing regulations such as the Solid Waste Management Act 2011, Solid Waste Management Rules 2013, and National Environment Policy 2019 encompass provisions related to plastic waste management. Key mechanisms include the 2021 ban on the use of plastic bags and the prohibition on the use of plastic flowers, serving as significant regulatory measures to control and manage plastic waste in Nepal [8]. Despite numerous rules and directives aimed at banning plastic bags, Nepal faces implementation challenges.

Nepal currently lacks specific regulations addressing the issue of microplastic pollution, particularly concerning water quality standards for drinking, aquaculture, and the presence of microplastics in various food items (Salt, sugar, milk, freshwater fish). This regulatory gap poses significant challenges in monitoring and mitigating the impact of microplastics on water sources and food safety within the country. The absence of guidelines specifically addressing microplastic pollution emphasizes the need for comprehensive measures to assess, control, and establish standards for microplastics in drinking water and food items. Bridging this regulatory void is crucial to safeguard public health, ensure environmental sustainability, and address the emerging challenges posed by the pervasive issue of microplastic pollution in Nepal.

The present utilization of plastics exhibits a lack of reuse, resulting in the generation of a substantial quantity of plastic waste that ultimately enters the natural environment. A significant portion of plastic waste is usually collected along with other municipal solid waste materials, in which plastics constitute a substantial component [9].

Plastic pollution has emerged as a significant global concern, particularly in the context of the COVID-19 pandemic. The extensive utilization of personal protective equipment (PPE), including disposable face masks, the rise in the consumption of takeaway plastic containers and utensils from restaurants, and the increased online shopping activities have collectively contributed to a sharp generation of plastic waste [10,11]. The amount of mismanaged plastic debris is projected to reach over 270 million metric tons if there is no improvement in present waste management techniques by the year 2060. This rise in waste would primarily occur in nations with less developed waste management systems [9]. Plastic waste experiences alterations in its physical properties through abiotic and biotic degradation processes such as heat, UV radiation, mechanical stress, enzymatic oxidation, and transformation into microplastics [12].

The term “microplastics” was first introduced by Thompson et al. [13] to refer to the aggregation of microscopic plastic fragments seen in marine sediments and the water column in European waterways. Arthur & Baker [14] introduced a proposed upper size threshold for the initial term “microplastics,” which referred to plastic particles that are smaller than 5 mm. Microplastic particles are typically divided into two categories: primary particles, which are manufactured for specific reasons, and secondary microplastic particles, which are formed as a result of the fragmentation of larger plastic components [15]. Microplastics occur in a variety of shapes and sizes, including fibers, fragments and spheres. The majority of microplastics result from the breakdown of larger plastics called macroplastics. Microplastics degrade into ever smaller debris over time, eventually transforming into nanoplastics (less than 1 μm) [16]. As a result, microplastics basically represent a transitional condition between macro debris and nanomaterials. Studies focusing on nanoplastics and nano waste particles with dimensions in the nanometer range have gained momentum in recent years. These investigations explore into the minute scale of plastic pollution and its potential implications [17,18]. The process of plastic fragmentation occurs mainly in terrestrial environments, which can be attributed to the higher ambient temperatures, increased frictional forces, and exposure to ultraviolet (UV) radiation [19].

Microplastics is present now everywhere from the bottom of the sea to top part of Mount Everest. The abundance of microplastics is recorded in snow and stream water of Mount Everest [20]. Microplastics have been detected in air [21,22], soil [23], lake water [24], lake sediments [25], beach sediments [26], river water [27], Ships ballast water and seawater [28], table salt [21], drinking water [29], glaciers [30], human stool [31], sewage sludge [32], fishes [[33], [34], [35], [36]], food items [37] and many living creatures. Microplastic pollution is distributed across the atmosphere, hydrosphere and pedosphere [38].

The investigation conducted by the majority of researchers involved the examination of distinct types of polymers in samples. There are six primary categories of microplastics that are distinguished by their chemical composition and density. These categories encompass polypropylene (PP), polyvinyl chloride (PVC), polyethylene terephthalate (PET), polystyrene (PS), high-density polyethylene (HDPE) and low-density polyethylene (LDPE). In general, plastic polymers, including polyvinyl chloride and polyethylene terephthalate, which possess a higher density (1.02 g/mL) than saltwater, exhibit a tendency to concentrate in sediments. Moreover, the concentration of these polymers increases in close proximity to heavily inhabited human areas [26]. Yang et al. [27] reported primary contributors to microplastic pollution in the river were point sources linked to predominantly raw sewage and solid waste originating from the municipal sectors. Microplastics are also derived from non-identifiable sources which might be transport and deposition from atmosphere and farmlands. The pollution can be attributed to various factors, including urbanization, agricultural activities, traffic, and tourism and fishing activities [27].

The Himalayan region attracts a significant number of both domestic and international tourists throughout the year. Consequently, this large number of visitors leads to the accumulation of a substantial and unmanageable quantity of plastic waste, which ultimately finds its way into the natural environment, including forests, streams, and rivers. The process of fragmentation of plastic waste has the potential to result in the production and subsequent buildup of microplastics in the Himalayan region [39]. Recently, Stefánsson et al. [30] reported the presence of microplastics in the glacier and suspected that the presence of microplastic particles within ice could potentially impact the melting process and rheological properties of glaciers. Consequently, this influence may have implications for the future contribution of meltwater to the oceans and the subsequent rise in sea levels. The results of their study found that the transportation of microplastic particles in the atmosphere is a significant route for the dissemination of microplastic pollution in glaciers. The accumulated plastic waste, often trapped in flooded forests, undergoes degradation, forming microplastics. These microplastics can integrate into the soil or re-enter the water, posing a new threat to Amazonian ecosystems [40]. According to Talbot & Chang [41], the spatial distributions of microplastics are significantly impacted by human activities, particularly in areas with urban land cover, dense populations, and the discharge of wastewater treatment plants.

The widespread recognition of the prevalence of microplastics in both land and aquatic ecosystems, along with the associated ecological consequences, has led to a greater realization of the potential risks posed to human exposure [42]. Microplastics are particles that range in shape, size, and composition rather than being discrete compounds or clearly defined substances. In general, plastic polymers are believed to be low in toxicity. Due to insoluble nature of microplastics, they are unlikely to be absorbed from the gastrointestinal tract and normally do not interact with biological matrices. However, may contain additives and unbound monomers that, in some cases, could leach out into the gastrointestinal tract and become bioavailable or into the surrounding aquatic environment before being consumed by humans. Furthermore, environmental pollutants, some of which are toxicologically problematic, can be absorbed by plastic particles [29]. Plants also have the capacity to absorb and transport micro/nanoplastics, leading to their accumulation in various tissues [43].

Microplastic is also reported in food items. The presence of microplastics in table salt and drinking water has the potential to be ingested by humans through the digestive tract. Similarly, microplastics in the air can lead to exposure of both the digestive and respiratory systems. Suspended microplastics have the potential to be inhaled, whereas deposited microplastics can be swallowed by hand-to-mouth contact, particularly among children [22]. The study examined the human body loads of microplastics resulting from the consumption of table salt, drinking water, and inhalation to range from 0 to 7.3 × 10^4^, 0 to 4.7 × 10^3^, and 0 to 3.0 × 10^7^ items per person per year, respectively. The inhalation of microplastics, particularly through indoor air, exhibited significantly greater levels of intake compared to other means of exposure. In addition, the presence of microplastics in the atmosphere poses potential risks to respiratory and digestive systems as a result of inhalation and ingestion [21]. The presence of microplastics in Himalayan lakes and rivers has become a concerning issue, as these water bodies are being subjected to contamination. The presence of microplastics has the potential to impact both the structure and function of the ecosystems in the Himalayan region. The presence of microplastics has been found to be associated with adverse health effects such as cancer, chromosomal damage and infertility within the local population [39].

Microplastic study in Nepal is in an early stage; hence the purpose of this review is to provide a concise overview of microplastic study and encompassing its characteristic features of microplastics. Moreover, it intends to outline the present state of microplastic contamination and offer prospective recommendations specific to the context of Nepal.

## 2 Methodology

In order to achieve the objective of the study, initially, keywords were employed in the most popular searching database such as Web of Science, Scopus and Google Scholar. The keywords used in this review were: Microplastics AND Nepal OR Microplastic pollution AND Nepal, Microplastic AND Freshwater Nepal OR Microplastics AND River Nepal, OR Microplastics AND Lake Nepal, OR Microplastics AND Riverine Ecosystem Nepal. Initially, the search results were compiled manually in a spreadsheet, and then any duplicate entries were eliminated. To identify each study, significant findings were collected based on the research objective.

## 3 Results and discussion

### 3.1 Microplastics study in Nepal

Microplastics research in Nepal is a growing area of interest due to the increasing concern about plastic pollution in the environment. Researchers in Nepal are beginning to study the presence of microplastics in various ecosystems, including rivers, stream, lakes, snow and terrestrial environments.

**Fig. 2:**
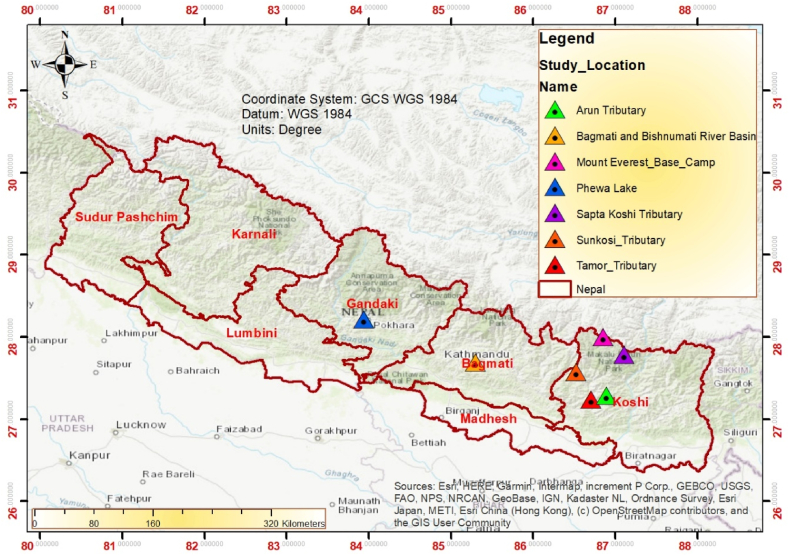
Microplastic research in Nepal showing different locations.

Microplastic study in Nepal is infant stage. Very few researches were conducted and limited in river, lake, snow and sediments. No any single paper has been found regarding the effects of microplastics on plant, animals, and human beings. The study on microplastics started in Nepal only from 2020. Only 6 papers were recorded from 2020 to 2023 (popular databases such as Web of Science, SCOPUS and Google Scholar). Two studies were conducted on lake sediments, and one each from river, Snow/stream and road dust (Table 1) (Fig. 2). Despite having a greater number of microplastics research in South Asian countries, Nepal had only six articles published. The review conducted by Kumar et al. [44] found that there has been a noticeable rise in research concerning microplastics in Asia starting from 2014. Particularly, there has been a substantial rise in the volume of studies conducted in 2018, and this trend continued to grow in 2021, with the number of scientific investigations quadrupling in comparison to the figures recorded in 2018. The findings revealed that although extensive study has been carried out in numerous Asian countries, the distribution of these studies was not uniform, as multiple investigations focused on certain rivers and lakes. Moreover, over two-thirds of the articles under consideration were centered on research conducted in China, with India and South Korea ranking as the subsequent focal points. According to Bayar et al. [45], Pakistan has only published two articles about microplastics.

**Table 1:** Microplastics Research in Nepal, examining different environmental components.

S.no	Title of Microplastics	Name of journal for publication	Studied environmental components	References
1	Microplastic pollution in lakeshore sediments: the first report on abundance and composition of Phewa Lake, Nepal	Environmental Science and Pollution Research	Lakeshore Sediments	[46]
2	Occurrence and Distribution of Microplastics from Nepal's Second Largest Lake (Phewa Lake)	Water, Air and Soil pollution	Shoreline sediments	[25]
3	Microplastic pollution in urban Lake Phewa, Nepal: the first report on abundance and composition in surface water of lake in different seasons	Environmental Science and Pollution Research	Lake/Surface water	[24]
4.	Microplastics in the Koshi River, a remote alpine river crossing the Himalayas from China to Nepal	Environmental pollution	River (water and sediments)	[27]
5.	Reaching New Heights in Plastic Pollution— Preliminary Findings of Microplastics on Mount Everest	One earth	Snow and stream water	[20]
6.	Occurrence and characteristics of microplastics in surface road dust in Kusatsu (Japan), Da Nang (Vietnam), and Kathmandu (Nepal)	Environmental pollution	Road dust	[47]

Similarly, a review of literature was undertaken to evaluate the existing status of microplastics research in ASEAN nations. The scholarly contributions from ASEAN countries constituted approximately 5 % of the global research papers on microplastics, with Indonesia being the largest contributor, succeeded by Malaysia and Thailand [48].

Over the past two decades, a large number of research has been conducted on the issue of microplastic contamination and its impact on many organisms across diverse regions globally. Amrutha et al. [49] in their study, provided a comprehensive examination of the microplastic research conducted by the member states of the South Asian Association for Regional Cooperation (SAARC), namely Afghanistan, Bangladesh, Bhutan, India, Maldives, Nepal, Pakistan, and Sri Lanka. A comprehensive analysis of 60 research papers revealed that India has made substantial contributions in the field of study under investigation. Bangladesh, Maldives, Nepal, Pakistan and Sri Lanka have reported a limited number of studies. The existence and impacts of microplastics in the environment of Afghanistan and Bhutan have not been reported so far.

The distribution of microplastics in oceans has received significant attention in recent years, with numerous studies conducted on this topic. These studies have demonstrated that microplastics are becoming a prominent form of pollution in marine environments, causing a threat to marine life [50,51]. A significant proportion of microplastic investigations in India has centered on marine environments, particularly the southeast coast, with a concentration of studies in Tamil Nadu. Sediment analysis has received greater attention in studying microplastic contamination within marine systems. Regarding freshwater systems, rivers have been the primary focus of studies compared to lakes and groundwater [52]. The study of microplastic pollution in Africa began recently compared to other continents. In 2016, the initial investigation into microplastic pollution in the African Great Lakes, specifically focusing on Lake Victoria, Nile perch, and Nile tilapia, was documented by Biginagwa et al. [53].

### 3.2 Methodology used for microplastic detection from various environmental components

Microplastic detection involves a comprehensive process encompassing various crucial steps, including sampling, extraction and identification (Table 2).

**Table 2:** Methodology used for Microplastics detection including Quality Assurance and Quality Control (QA/QC).

Sampling	Extraction	Identification	QA/QC	References
For each location, a 25 cm × 25 cm quadrat was utilized to collect top 2 cm shoreline sediments with a stainless-steel spoon. Pooled in a steel container, two replicates were sealed in aluminium foil bags, labeled for laboratory analysis.	Samples were dried at 60 °C for 24 h, weighed, and sieved (5 mm) to remove debris. In a beaker, 100 g sediment mixed with 400 mL saturated salt solution (undisturbed for 5 min). Floating solids passed through a 0.2-mm sieve, transferred to a beaker, and rinsed. After drying at 90 °C for 24 h, samples were weighed. Subsequently, 20 mL 30 % H_2_O_2_ and 20 mL 0.05 M Fe (II) solution were added for organic matter reduction, heated at 50 °C for 30 min. Density increased with NaCl, left for 24 h, supernatant filtered through a 1-mm sieve, and the solution filtered through Whatman GF/C filter paper. The filter paper was air-dried for 24 h for further examination.	The plastic caught in the 1-mm sieve underwent multiple washes with pure water, then carefully moved to a clean petri dish. Visual examination under a stereomicroscope (SZ2-ILST, Olympus, Japan) at 40 × magnification identified suspected microplastic particles. Polymer identification of microplastics in the size class of 1–5 mm was conducted using ATR-FTIR (IRAfnity, 1S, SHIMADZU, serial number A221352), capturing wavelengths in the range of 400–4000 cm−1.	Glassware was rinsed in Milli-Q water, covered with aluminium foil during processing. Wore lab coat and gloves for analysis. Filters stored in clean petri dish, examined for airborne contamination under a stereomicroscope during sediment analysis.	[46]
Collected the top 2 cm of shoreline sediment using a steel spoon from a 25 cm × 25 cm quadrat at each sampling point and stored in an aluminium foil container for subsequent analysis.	A beaker with 100 g sediment and 400 mL saturated sodium chloride solution was stirred, settled, and transferred to a clean beaker. After isolation, 20 mL each of 30 % hydrogen peroxide and 0.05 M ferrous sulfate were added, and the mixture was boiled at 50 °C. Following 24 h of density separation, the sample undertook sieving, and the filtrate passed through Whatman glass fiber filter paper	The air-dried filter paper was visually inspected under a stereomicroscope at 40 × magnification after 24 h. The chemical composition of suspected visible microplastic particles (size 1–5 mm) was analyzed using FTIR (IRAfnity-1S, SHIMADZU, serial number A221352) in the wavelength range of 500–4000 cm−1.	Used nitrile gloves, cotton coat, pre-washed tools, and glassware to prevent contamination during sampling and laboratory analysis. Filters were kept in a petri dish after use. Air blanks were kept to check for possible contamination	[25]
Collected 5 L surface water (0–20 cm) using a steel bucket, sieved through a 75-μm brass filter on-site, transferred to a glass bottle, and stored at 4 °C for analysis.	Treated water sample with 20 mL 30 % H_2_O_2_ and 20 mL Fe solution. Heated for 5 min at 50 °C, boiled, and kept for 15 min. Filtered through 1-mm sieve, then through 47-mm glass microfiber filter. Rinsed beaker, dried filter paper at room temperature for examination.	Examined the petri dish and filters visually under a stereomicroscope (SZ2-ILST, Olympus, Japan) at 40 × magnification. Identified microplastics based on morphological characteristics such as shape and color.	Use of cotton lab coat and nitrile gloves. Used glassware rinsed with Milli-Q water. Covered samples and glassware with aluminium foil to minimize contamination. After filtration, stored filter paper in a petri dish. Checked for airborne contamination under a stereomicroscope and left open in the working environment for 24 h. Conducted field blanks in four random sampling locations.	[24]
Surface water and sediment samples were obtained from each sampling site, eight sub-samples of 5 L surface water were collected from both river banks, filtered through a 100 μm stainless-steel sieve, and washed with filtered pure water. The contents were transferred into a 50 mL glass bottle and fixed in 5 % formalin at 4 °C. Additionally, eight sub-samples of 1 kg surface sediment were collected at each site (stainless-steel shovel), mixed thoroughly, and 1 kg of the mixed sediment was stored in a clean aluminium box at 4 °C. Prior to analysis, the sediment was dried for 72 h at 50 °C to achieve a constant mass.	Water samples were treated at 75 °C with 10 mL of 30 % H_2_O_2_ for 12 h, followed by filtration through a 0.45 μm filter. Filters were air-dried for analysis. Microplastics were extracted from sediment using density separation and ZnCl_2_ solution. After mixing dry sediment with ZnCl_2_, the solution was settled, and the upper layer was transferred. This process was repeated thrice for each sample. Hydrogen peroxide and Fe (II) solution were added to degrade organic matter, followed by filtration through a 0.45 μm filter. The filters were air-dried for microscopic inspection.	Microplastics were examined under a stereo-microscope (NIKON SMZ800 N, Japan) with an Olympus digital camera (DP26). Microplastic particles, representing common characteristics, were selected for polymer type analysis using μ-FTIR (Thermo Fisher Nicolet™ iN™10) in transmittance mode.	Rigorous measures were taken during sample collection and lab analyses to avoid plastic contamination. Lab personnel wore cotton lab-coats, masks, and latex gloves. Liquids were filtered through a glass fiber filter (0.45 μm), and tools were washed with ultrapure water. Procedural blanks were employed to monitor background contamination from lab sources.	[27]
900 mL stream water, 300 mL snow samples were kept in stainless steel containers.	In the lab, melted snow and stream water were vacuum-filtered onto Whatman 1.6 mm glass microfiber filter papers, and the filtered volume was noted. Examination was carried out using an S9E - Leica light microscope.	Suspected microplastics (>30 mm) were extracted from the filter paper and placed in glass petri dishes. Verification was done using Fourier-Transform Infrared Spectroscopy (FTIR) microscopy in transmission mode, utilizing a Hyperion 1000 microscope coupled to a Bruker Vertex 70 spectrometer.	Sample bottles, cleaned in the lab with Milli-Q water through Whatman 1.6 mm glass microfiber filters, were filled using ungloved hands from running streams or snow. Samples were promptly sealed and stored in a box within a sampling storage tent at Everest Base Camp (5364 m.a.s.l.) to minimize plastic contamination.	[20]
Collected road samples using a Makita vacuum cleaner (CL102DW) for 1 min at each 2 m × 5 m sampling site (10 m^2^). Individual disposal paper bags were used, and samples, stored in the dark, were immediately taken to the lab for pretreatment and analysis	Samples underwent initial sieving on a stainless-steel sieve (<100 μm mesh, 75 μm mesh). Treatment included a week-long exposure to 30 % hydrogen peroxide, drying at 50 °C, and 3-h treatment with 5.3 M aqueous NaI for specific gravity separation.	Stereoscopic microscope and digital camera (STZ-161-TLED, Moticam U, Shimadzu). Each collected microplastic piece underwent analysis using an attenuated total reflectance (ATR) - Fourier transform infrared spectrometer (Cary 630 FTIR, Agilent)	Laboratory Blank test	[47]

The initial phase centers around collecting samples from diverse environmental components such as lakeshore sediments, lake water, river water, snow, stream water, and road dust. The selection of sampling locations and methods is designed to capture a representative range of environmental conditions. Following the sampling process, the next step is to extract microplastics from the collected materials. This step involves specific procedures like filtering water samples, sieving sediments, or employing other techniques designed to suit the characteristics of the targeted environmental component. Finally, microplastics are identified through the utilization of advanced techniques, including microscopy methods like stereomicroscopy and Fourier-Transform Infrared Spectroscopy (FTIR).

### 3.3 Characteristics of microplastics detected in Nepal

Microplastics exhibit diverse characteristics in color, shape, size, and abundance, contributing to their complexity in environmental systems.

#### 3.3.1 Color

The role of color is of considerable importance in the potential categorization of microplastics when observed under a stereomicroscope. Transparent, red, blue and black colored microplastics were recorded from collected samples at this review (Table 3). One review conducted in Latin America, by Kutralam-Muniasamy et al. [54] showed among the 78 studies examined, a significant number of microplastics were found to exhibit different colors, such as transparent, white, blue, and a range of colored variants including green, pink, yellow, purple, violet, brown and black. The color of microplastics can vary due to several factors, and different colors are often attributed to factors like composition, production methods, and environmental exposure. Microplastic color is useful for identifying polymer types; clear, transparent ones are typically polypropylene (PP), while white and light-colored ones are often polyethylene (PE) and low-density polyethylene (LDPE). Surface wear and fading on microplastics can indicate the extent of weathering [55]. Manufacturers incorporate additives and dyes into plastics for various purposes, contributing to microplastic coloration. Environmental exposure, such as sunlight and water, can cause degradation and weathering, altering microplastic color over time. The original plastic purpose and source can also influence color, and aging processes can impact color through changes in molecular structure [56].

**Table 3:** Physical and chemical attributes of microplastics.

S no	components	Color	Shape	Size	Abundance	Type of polymers	References
1	Lakeshore sediments (Phewa Lake)	Transparent followed by red	Fibers (78.11 %)	0.2–1 mm size (70.65 %)	100.5 ± 58.6 items/kg dry weight	Polypropylene (PP) (42.86 %) followed by polyethylene (PE)	[46]
2	Shoreline sediments (Phewa Lake)	transparent (23.53 %) blue (21.39 %).	Fibers 62.03 %	0.2–1 mm (highest)	Range in between 55 and 122.5 items/kg dry weight	Polypropylene (PP) and polyethylene (PE)	[25]
3	Lake water (Phewa Lake)	transparent (both seasons)	Fibers 96.7 % (wet season) (93.04 % winter)	<1 mm in size	2.96 ± 1.83 MP/L in winter and 1.51 ± 0.62 MP/L in wet season	–	[24]
4	Water and sediments (Koshi River)	Blue and black	Fibers (98 %)	small MPs (<1 mm) accounted for approximately 60 % of all MPs.	Mean abundances of microplastics in water and sediment were 202 ± 100 items/m3 and 58 ± 27 items/kg, dry weight, respectively	Polyethylene (PE), polyethylene terephthalate (PET), polyamide, polystyrene (PS) and polypropylene (PP)	[27]
5	Snow/Stream water (8440 m.a.s.l)	**-**	Fiber	<5 mm	30 MP/L (snow) 1 MPs/L (stream water)	Polyester	[20]
6	Road dust	Varieties of colors	Fragments	**-**	12.5 ± 10.1 pieces/m2	**-**	[47]

#### 3.3.2 Shape and size

The determination of particle size is of utmost importance due to its significant impact on the transport dynamics and environmental fate of microplastics in aquatic ecosystems, as well as its influence on interactions among various aquatic animals. The size distribution of microplastics was documented in all studies related to aquatic bodies. The dominant size of microplastic in this review is less than 1 mm recorded (Table 3). Similar observation was reported in drinking water and other food items that has been found to include a significant quantity of microplastics measuring less than 1 mm [54,57]. Sewage sludge is also rich in microplastics which was reported by Ren et al. [32]. The findings of Ren et al. [32] indicated that the observed microplastic particles exhibited a size distribution ranging from 8 μm to 1 mm, with a significant proportion falling within the range of 8–400.00 μm, which corresponds to 97.27 % of the total.

#### 3.3.3 Abundance

Various researchers reported that the abundance of microplastics vary from types of samples and locality. The freshwater microplastic distribution is influenced by the spatial and temporal scale at which it is examined. Certain hydrological and anthropogenic processes have the potential to exert significant influence on microplastic abundance within specific spatial and temporal scale [41]. The abundance of microplastics is subject to influence from intrinsic factors associated with the varying seasons, specifically in relation to precipitation [58]. The process of precipitation has the potential to facilitate the transfer of microplastics originating from land to aquatic ecosystems. Notably, studies conducted by Schmidt et al. [59] Wong et al. [60], and Xia et al. [58] have reported significant increases in the presence of microplastics in surface waters subsequent to rainfall events. Sea water is the main reservoir for the microplastic abundance. One recent study conducted by Zendehboudi et al. [28] reported that Ballast water transport is a major conduit for the global spread of various pollutants, including microplastics, across the world's oceans. The average concentrations of microplastics in ballast water and seawater samples were 12.53 and 11.80 items per liter, respectively, with the predominant size category being 50–300 μm.

The abundance of microplastics in various environmental components in Nepal reflects a complex and intricate situation. Lakeshore sediments, a critical ecological interface, exhibit a notable concentration of 100.5 ± 58.6 items/kg dry weight, suggesting a significant microplastic presence in these aquatic ecosystems [46]. Along shoreline sediments of Phewa lake, the variability in concentrations ranging between 55 and 122.5 items/kg dry weight [25] emphasizes the diverse nature of areas and their distinct microplastic loading. In the context of lake water, a seasonal fluctuation is observed, with 2.96 ± 1.83 MP/L in winter and a comparatively lower concentration of 1.51 ± 0.62 MP/L in the wet season [24]. This seasonality may indicate potential influences of weather patterns on microplastic transport and deposition. The Koshi River, major river of Nepal, demonstrates a higher abundance in water (202 ± 100 items/m^3^) than in sediments (58 ± 27 items/kg dry weight), emphasizing the dynamic interplay between flowing water and sedimentary deposition [27]. Further insights into atmospheric transport emerge from the snow component, registering a concentration of 30 MP/L, signifying the role of atmospheric deposition in introducing microplastics into the environment. In contrast, stream water displays a lower concentration of 1 MP/L, indicating potential differences in pollution sources or transport dynamics [20]. The microplastic load in road dust provides an additional layer of complexity, with a concentration of 12.5 ± 10.1 pieces/m^2^ [47] This underlines the varied pathways through which microplastics enter different ecosystems in Nepal, highlighting the need for comprehensive studies to understand the intricate dynamics of microplastic pollution and its implications for environmental health. (Table 3).

#### 3.3.4 Types of polymers

The identification of various polymers within the environmental components of the study was conducted by researchers using Fourier-Transform Infrared (FTIR) methods, allowing for the precise characterization and differentiation of distinct polymer types present in the sampled materials. The study conducted in Nepal documented the presence of various types of polymers in both water and sediments (Table 3). These polymers encompassed polypropylene (PP), polyethylene (PE), polyethylene terephthalate (PET), polyamide (PA), polystyrene (PS), and polyester. In the lakeshore sediments of Phewa Lake, a crucial aquatic interface, the primary polymer identified is Polypropylene (PP), constituting a significant proportion of 42.86 %, followed closely by Polyethylene (PE) ecosystems [46]. Similarly, in the shoreline sediments of Phewa Lake, the polymers present include Polypropylene (PP) and Polyethylene (PE), indicating a distinct composition compared to lakeshore sediments. Polypropylene (PP) and Polyethylene (PE) emerge as predominant polymers in plastic production, collectively constituting 50.0 % of the European plastic demand, with Polyethylene accounting for 30.3 % and Polypropylene for 19.7 % [61]. This dominance is consistent with findings in various studies, including those by Irfan et al. [62], Klein et al. [63] and Rodrigues et al. [64] which consistently identify PE and PP as the most prevalent plastic particles in sediments. Polypropylene (PP) finds extensive application in daily life, particularly in food packaging and the wrappers of sweets and snacks [61]. The water and sediments of the Koshi River, a major water body in Nepal, exhibit a broader spectrum of polymers, encompassing Polyethylene (PE), Polyethylene Terephthalate (PET), Polyamide, Polystyrene (PS), and Polypropylene (PP). Polyethylene (PE) and polypropylene (PP) constituted the major polymer types, representing 38 % and 30 % of the total microplastics in water. The detection of these polymers in Nepal may be linked to significant production in China and the export of plastic from China to Nepal. In sediments, Polyethylene emerged as the predominant polymer, accounting for 44.26 % of the total, followed by polyamide (PA or nylon) at 32.79 %, PET at 16.39 %, and PS at 6.56 % [27]. In Mt. Everest samples, the identified polymers predominantly include polyester (56 %), acrylic (31 %), nylon (9 %), and polypropylene (5 %) [20]. These polymers align with the materials commonly used in outdoor gear manufacturing. Polyester, acrylic, and polypropylene are standard fibers employed in clothing for outdoor activities. Additionally, polyester and nylon are popular choices for manufacturing tents and climbing ropes [65], emphasizing the prevalence of these materials in the equipment utilized in high-altitude environments like Mt. Everest).

The study conducted in Latin America by Kutralam-Muniasamy et al. [55] included polyethylene, polypropylene, polyethylene terephthalate, and polystyrene as the predominant polymer types, collectively constituting 80 % of the overall polymer types. Ren et al. [32] observed 41.18 % of the microplastics in sewage sludge were specifically recognized as polyvinyl chloride (PVC), hence contributing to an elevated risk rating. The predominant polymer types seen in the aquatic environment and biota were polyethylene (PE), polypropylene (PP), polyethylene terephthalate (PET), and polystyrene (PS) [[66], [67], [68]] These four polymer types collectively constituted approximately 80 % of the total polymers present. The particles composed of polyethylene (PE) were the most frequently documented, accounting for 40 % of the overall quantity [54].

### 3.4 Opportunities, challenges and current gaps in knowledge of microplastics study in Nepal

#### 3.4.1 Opportunities and challenges- research grant funding organization in Nepal

Studies in Nepal receive funding from diverse sources, including government agencies, international organizations, non-profits, and research institutions. The Nepalese government actively supports research through grants and collaborations. Strategies to combat issues like environmental pollution/plastic pollution involve regulatory enforcement, public awareness campaigns, and investments in sustainable waste management, often in collaboration with NGOs, private sectors, and international partners.

Nepal Academy of Science and Technology (NAST) is a governmental institution that support scientific research in various field. NAST annually awards research grants to Nepali professionals, aiming to promote scientific research within the country. The program prioritizes research with a focus on local resources and addressing identified needs. Researchers, scholars, and scientists affiliated with academic institutions in Nepal are typically eligible to apply for NAST grants. The eligibility criteria may vary based on the specific grant program [69].

University Grants Commission (UGC) Nepal is another active body which allocates funds to support academic research projects proposed by faculty members and researchers affiliated with universities and academic institutions in Nepal. These grants are typically aimed at advancing knowledge in diverse fields, fostering innovation, and contributing to national development. UGC Nepal may identify specific research priority areas aligned with national development goals. Researchers are encouraged to submit proposals that address challenges and contribute to solutions in these priority areas. UGC Nepal has allocated Nepalese Rupees (NPR) 2,00000/- for Small Research Development and Innovation Grants, NPR 4,00000/- for faculty research grants and NPR 20,00000/- for collaborative research grants. Collaborative research grants by UGC aim to foster partnerships between researchers and institutions. These grants encourage joint efforts to address research challenges and contribute to knowledge advancement [70].

Likewise, Research Centre for Applied Science and Technology (RECAST), Centre for Nepal and Asian Studies (CNAS), and Tribhuvan University Rector's Office offer research grants to incentivize educators and teaching professionals in pursuing research endeavors. RECAST aims to form collaborations with national and global academic institutions, government bodies, private sectors, and community-based organizations. RECAST is committed to supporting the nation in reaching Sustainable Development Goals (SDGs) and advancing to the status of a developing country [71].

However, the research advancement in Nepal remains unsatisfactory despite the presence of organizations offering research grants. The comparatively sluggish progress of research in Nepal, in contrast to other global regions, can be attributed to multifaceted factors. Insufficient research infrastructure, limited funding, and resources pose significant challenges, along with inadequacies in the education system that hinder a robust research culture. Periods of political and economic instability contribute to disruptions, and the lack of effective collaboration between academia, industry, and government further impedes holistic research development. Brain drains, regulatory hurdles, and a focus on immediate concerns such as poverty and healthcare prioritize short-term goals over long-term research initiatives. Global disparities in access to research networks and technologies also put Nepalese researchers at a disadvantage.

The field of microplastics research in Nepal is still in its infancy. There is a lack of awareness about this microplastics issue among the government, universities, research institutes, research grant organizations, and researchers in the country. Consequently, essential equipment like Stereomicroscopes and FTIR (Fourier-Transform Infrared Spectroscopy) devices are very limited and not readily accessible in research organizations and reputed laboratories. The high cost associated with testing for microplastics further discourages researchers from investigating into this area of study. This overall lack of awareness, limited availability of crucial equipment, and the financial constraints pose significant challenges, hindering the progress of microplastics research in Nepal.

Nepal possesses abundant freshwater resources on a global scale. The region is characterized by the presence of around 6000 rivers and several lakes. To date, research on microplastics has focused only on the study of a single lake and a single river. Hence, Nepal presents ample opportunities for conducting extensive studies (Table 4). Engaging in research on a novel subject matter of microplastics enables to make a substantial contribution to the new body of knowledge. It possesses the inherent capacity to uncover novel perspectives and resolutions that might provide advantageous outcomes for the broader community. Pioneering research in microplastics can enhance career prospects, increase visibility in the environmental field, and lead to academic or professional recognition.

**Table 4:** Possible topics for microplastics research in Nepal.

Sno	Possible topics for microplastics research
1	Investigating the presence and extent of microplastics in the water supply, including bottled water, to assess potential human exposure.
2	Examining the prevalence of microplastics in different food products consumed in Nepal, evaluating the implications for human health.
3	Assessing the occurrence of microplastics in organic fertilizers, exploring potential implications for soil health and crop contamination.
4	Investigating the physiological and health impacts of microplastic exposure on the Nepalese population.
5	Studying the consequences of microplastics on bird species in Nepal, evaluating potential ecological and behavioral impacts.
6	Analyzing the impact of microplastics on diverse wildlife species in Nepalese ecosystems, addressing potential ecological consequences.
7	Examining the influence of microplastics on pets and livestock, considering potential health and environmental implications.
8	Investigating how microplastics may affect soil quality, crop growth, and food safety in agricultural settings.
9	Assessing the impact of microplastics on freshwater fish species, examining potential ecological and human health concerns.
10	Exploring the accumulation and magnification of microplastics through different trophic levels in Nepalese ecosystems.

#### 3.4.2 Current gaps in knowledge

• Nepal, characterized by its majestic mountainous terrain adorned with numerous Himalayan peaks, including the iconic Mount Everest, presents a unique and challenging landscape for environmental research. There is a compelling need to scientific investigations across the diverse ecological spectrum, ranging from upstream Himalayan River sources to the downstream rivers in the Terai region of Nepal. Currently, there is a notable lacking of information regarding the abundance, distribution, and environmental impacts of microplastics in these critical areas. Similarly, the occurrence of microplastics study on glacier and glacial lake is very limited on a global scale. Nepal has many glacial lake and glaciers thus there is urgent need of microplastic distribution the country.
• The prevalence of aquatic microplastic pollution has been documented in Nepal, however, there remains a lack of knowledge regarding airborne microplastics. Urban areas in Nepal, especially in the Kathmandu Valley, facing with severe air pollution characterized by elevated levels of various pollutants. Despite extensive monitoring of major parameters across different regions of Nepal, the examination of microplastics and their potential impacts has received limited attention. A comprehensive study focusing on the presence, sources, and effects of microplastics in urban air environments, especially in the context of Nepal's unique geographical and environmental conditions, would significantly contribute to addressing this critical gap in current research efforts.
• Current microplastic pollution research tends to utilize snapshot techniques, focusing on specific moments and limited regions. To advance understanding a shift towards long-term (seasonal changes in both lentic and lotic ecosystem), regionally extensive investigations is vital. This comprehensive approach should involve continuous monitoring of microplastic dynamics, allowing for clear insights into abundance, distribution, and ecological impacts over time.
• The field of toxicological research pertaining to the effects of microplastics is now in its early stages. Ongoing research studies in this field, however, have begun to reveal some common features. However, it is imperative to periodically collect and evaluate this knowledge in order to derive conclusions and establish directions for future research. To date, there is an absence of comprehensive studies in Nepal regarding the presence and consequences of microplastic pollution on both terrestrial and aquatic biota. Source identification, movement, distribution, and fate of microplastics in both terrestrial and aquatic environments need to be thoroughly examined.
• Although few studies have been carried out on microplastic impact on human health on a global scale, the potential long-term health implications resulting from the consumption of microplastics through food are unclear at present. No research was found in Nepal regarding this matter.
• The expanding amount of information about the presence of microplastics in drinking water and other consumable products, such as soft drinks and beer, is cause for concern. Researchers in Nepal have the opportunities to study these foods.

## 4 Conclusion

Research on microplastics has received significant attention in recent years. The current state of microplastic research in Nepal is in its early stages. The examination of microplastics in Nepal began in the year 2020. A total of six papers were documented within the time frame of 2020–2023. A limited number of studies have been undertaken in the domains of rivers, lakes, snow, and sediments. Studies have demonstrated the widespread presence of microplastics in various aquatic environments, including surface waters (lakes and rivers), snow and sediments. The prevalence of microplastics in freshwater ecosystem is among the most well documented findings. In freshwater ecosystem, microplastics, specifically fibers, was found to be the prevailing form, while fragments were observed in road dust. The research undertaken in Nepal demonstrated the occurrence of diverse polymer varieties in both aqueous and lake sediments. The polymers included polypropylene (PP), polyethylene (PE), polyethylene terephthalate (PET), polyamide, polystyrene (PS), and polyester. While numerous studies have been conducted on the topic of microplastic contamination and its impact on organisms, no scholarly publications in Nepal have been identified that specifically examine the impacts of microplastics on animals, plants, and human. There is a noticeable deficit of studies examining the human consumption of microplastics and the possible hazards they pose to human health.

## Funding

This research did not receive any specific grant from funding agencies in the public, commercial, or not-for-profit sectors.

## CRediT authorship contribution statement

**Kishor Kumar Maharjan:** Writing – review & editing, Writing – original draft, Validation, Investigation, Formal analysis, Data curation, Conceptualization.

## Declaration of competing interest

The author declares that they have no known competing financial interests or personal relationships that could have appeared to influence the work reported in this paper.
